# Tuberculosis Management Practices of Private Practitioners in Pune Municipal Corporation, India

**DOI:** 10.1371/journal.pone.0097993

**Published:** 2014-06-04

**Authors:** Sandeep Bharaswadkar, Avinash Kanchar, Narendra Thakur, Shubhangi Shah, Brinda Patnaik, Eleanor S. Click, Ajay M. V. Kumar, Puneet Kumar Dewan

**Affiliations:** 1 Office of the World Health Organization (WHO) Representative in India, New Delhi, India; 2 Directorate of Health Services, Pune Municipal Corporation, Maharashtra, India; 3 Department of Community Medicine, MIMER Medical College, Talegaon, Pune, India; 4 Division of Tuberculosis Elimination, Centers for Disease Control and Prevention (CDC), Atlanta, Georgia, United States of America; 5 International Union Against TB and Lung Diseases, Regional Office for Southeast Asia, New Delhi, India; Johns Hopkins Bloomberg School of Public Health, United States of America

## Abstract

**Background:**

Private Practitioners (PP) are the primary source of health care for patients in India. Limited representative information is available on TB management practices of Indian PP or on the efficacy of India’s Revised National Tuberculosis Control Programme (RNTCP) to improve the quality of TB management through training of PP.

**Methods:**

We conducted a cross-sectional survey of a systematic random sample of PP in one urban area in Western India (Pune, Maharashtra). We presented sample clinical vignettes and determined the proportions of PPs who reported practices consistent with International Standards of TB Care (ISTC). We examined the association between RNTCP training and adherence to ISTC by calculating odds ratios and 95% confidence intervals.

**Results:**

Of 3,391 PP practicing allopathic medicine, 249 were interviewed. Of these, 55% had been exposed to RNTCP. For new pulmonary TB patients, 63% (158/249) of provider responses were consistent with ISTC diagnostic practices, and 34% (84/249) of responses were consistent with ISTC treatment practices. However, 48% (120/249) PP also reported use of serological tests for TB diagnosis. In the new TB case vignette, 38% (94/249) PP reported use of at least one second line anti-TB drug in the treatment regimen. RNTCP training was not associated with diagnostic or treatment practices.

**Conclusion:**

In Pune, India, despite a decade of training activities by the RNTCP, high proportions of providers resorted to TB serology for diagnosis and second-line anti-TB drug use in new TB patients. Efforts to achieve universal access to quality TB management must account for the low quality of care by PP and the lack of demonstrated effect of current training efforts.

## Introduction

According to the third National Family Health Survey in India, conducted in 2006, the private sector remains a primary source for health care for patients of all socioeconomic levels [1]. A large proportion of patients with tuberculosis (TB) in countries with high TB prevalence first seek care with a private practitioner [2]. In India, it is estimated that 46% of patients with TB seek care in the private sector [3]. However, TB diagnosis and treatment practices among private practitioners in India vary widely and are not formally regulated by the national TB programme [4].

In 2002 India’s Revised National Tuberculosis Control Programme (RNTCP) introduced guidelines for involvement of Private Practitioners (PP) through an initiative called ‘Public Private Mix’ which encompasses training activities and formal collaboration with the RNTCP [5]. RNTCP provides training and encourages PP to adhere to the International Standards of TB Care (ISTC) [6]. PP may also collaborate formally with the RNTCP by serving as a referral facility, diagnostic centre, or treatment centre after entering into a formal memorandum of understanding with the RNTCP.

Despite ongoing efforts to train PP on standard clinical practices, a recent non-representative survey from Mumbai suggests that practices of PP have not improved markedly from those of a decade earlier. [7] In Pune Municipal Corporation (PMC), the urban capital city of the South Indian State of Maharashtra, RNTCP training has been offered and promoted by RNTCP since 2003. However, the impact of efforts made by the RNTCP to influence the quality of TB care in the private sector in Pune remains largely unknown. We sought to assess the influence of RNTCP training on PP in Pune by comparing reported clinical practices relative to ISTC standards for providers based on whether or not they had been exposed to RNTCP training.

## Materials and Methods

### Study Design

Cross-sectional using a structured questionnaire (Questionnaire S1), in Pune, an urban area of western India in 2010.

### Study Participants

Private practitioners of allopathic medicine and those having a degree in any system of medicine (i.e., allopathy, homeopathy, Ayurveda or Unani) were considered eligible for participation in the study. Specialists who generally do not treat TB cases (e.g., psychiatrists) were excluded.

### Sampling

Names and contact information for PPs were provided by the Indian Medical Association, General Practitioner’s Association and National Integrated Medical Practitioner Association. Private practitioners were selected by systematic random sampling from a consolidated list of names of eligible participants (n = 3,391). We calculated a sample size of 246 respondents would be needed based on estimated parameters for exposure to RNTCP training programs and adherence to ISTC standards. If a practitioner had relocated from Pune Municipal Corporation area, was not currently practicing, or declined participation, the interviewers selected the next provider on the list of eligible participants. A maximum of two attempts were made to interview each participant.

### Data Collection

Study participants were interviewed in person using a pre-tested semi-structured questionnaire by one of seven trained interviewers. The questionnaire contained sample clinical case scenarios and responders were asked to describe their approach to diagnosis and treatment for each scenario. A combination of open-ended and multiple-choice questions was used.

### Definitions

*Exposure to RNTCP training* was determined by self-report by providers and was defined as participation in the 2002 RNTCP training module of at least 4 hours at any time from 2002 through 2010.

*‘Involvement with RNTCP’* was based on self-report by providers and was defined as having participated in at least one of the programs designed for incorporation of private practitioners in government TB control activities. Examples of involvement with RNTCP include: 1.) Use of RNTCP microscopy centers for diagnostic evaluation of patients, 2.) Provision of directly observed treatment (DOT) with drugs and supervision provided by the RNTCP, 3.) Referral of presumptive TB patients to RNTCP centers for diagnosis 4.) Referral of TB patients to local government treatment centers for treatment. Completion of a formal MOU with the RNTCP was not considered a requirement for involvement with RNTCP.

*Adherence to standards for diagnosis* was determined based on participant responses to the following three clinical scenarios: 1) presentation consistent with pulmonary TB without indication of previous TB treatment (new pulmonary TB), 2) presentation consistent with extra pulmonary TB, and 3) presentation consistent with pulmonary TB after successfully completing treatment for a previous diagnosis of TB (recurrent pulmonary TB). Providers were considered to have diagnostic practices in accordance with ISTC guidelines for each type of TB if their response to the corresponding scenario contained at least the following elements: 1) for new pulmonary TB: collect at least 2 sputum specimens for microscopy, 2) for extra-pulmonary TB: use bacteriology and histopathological examination for diagnosis, and 3) for recurrent pulmonary TB: use sputum culture and drug susceptibility testings [6].

*Adherence to standards for treatment* was determined for treatment scenarios consistent with treatment of a new case of pulmonary TB. For new cases of TB, providers were considered to have treatment practices in accordance with ISTC guidelines if their responses to the scenario contained use of only the four first-line anti-TB drugs: drugs: (Rifampicin (R), Isoniazid (H), Pyrizinamide (Z) and Ethambutol (E)) daily or three times per week for at least 6 months [6].

### Data Handling and Analysis

Our data were entered by two investigators separately and discrepancies were resolved by referring to the original data collection tool. We calculated the proportion of providers that adhered to international standards and stratified based on whether or not they had been exposed to RNTCP training. We calculated odds ratios to estimate the association between RNTCP training and adherences to international standards of TB care. We used 95% confidence intervals (CI) to assess the statistical significance of our estimates.

### Ethics Considerations

Approval for ethical and scientific review was obtained from the relevant committees of the International Union Against TB and Lung Diseases and National Tuberculosis Institute, Bangalore. Additional review by CDC institutional review board was not required because CDC investigators were determined not to be engaged in human subjects research as defined by relevant US government regulations (i.e., CDC investigators did not interact with study subjects or have access to identifiable data for study subjects). Administrative approvals were obtained from local state authorities ie State TB Officer, Government of Maharashtra and written informed consent was obtained from each participant.

## Results

Of 268 providers approached, 249 were interviewed (93% participation rate) (Table 1). Of these, 175 (70%) did not have an allopathic medical (Bachelor of Medicine & Bachelor of Surgery, [MBBS]) degree and 154 (62%) had more than 10 years of professional experience. 90% of PP had also evening hours clinic which was assessed to know relationship if any of clinic hours with exposure to training. A total of 136/249 (55%) had been exposed to RNTCP training and 142/249 (57%) were involved with RNTCP. More than 90% of Private Practitioners either work as referring centre or DOT centre. As a referral centre, they refer presumptive TB patients/TB patients for diagnosis/treatment.

**Table 1:** Characteristics of private providers participating in the study – Pune Municipal Corporation, India, 2010.

Characteristic	Total (N = 249)	%	(95% CI)
**Gender**			
Male	185	74	(69–80)
Female	64	26	(20–31)
**Medical Qualification**			
MBBS+ & above	74	30	(24–36)
Non-MBBS++	175	70	(65–76)
**Clinical Experience** +++			
≤10 years	95	38	(32–44)
>10 years	154	62	(55–67)
**Relationship with RNTCP**			
Involved	142	57	(32–44)
Not involved	107	43	(37–50)
**Exposure to Training**			
Exposed	136	55	(48–60)
Unexposed	113	45	(39–51)
**Clinic hours for outpatient consultation** †			
Includes evening clinic hours	232	94	(90–97)
No evening clinic hours	15	6	(4–10)

+ Allopathy doctor (Bachelor of Medicine and Bachelor of Surgery).

++ Doctors practicing homeopathy, Ayurveda, Unani.

+++ Number of years in clinical practice after completion of medical training.

† Information not available for two study participants.

Drugs (Patient wise boxes) are made available from programme to DOT centre. Patients come to DOT Centre and takes medicine under observation of Private Practitioners.

Less than 10% Private Practitioners are involved in other schemes like designated Microscopy centres or sputum collection & transport centres.

Proportion of PP with qualification other than MBBS participating in study were 70%. This proportion is similar to that in community where PP possessing qualification other than MBBS are more in number. In contrast to providers with MBBS degrees, providers with other medical qualifications were more likely to have been exposed to RNTCP training (OR 2.4, 95% CI 1.4–4.4).

Providers who are involved with RNTCP were more likely to have been exposed to RNTCP training than providers not involved with RNTCP (OR 5.1, 95% CI 2.8–9.1). There was no association between exposure to RNTCP training and provider sex, years of experience, or clinic hours of operation **(** **Table 2** **)**.

**Table 2:** Characteristics of private providers based on reported exposure to RNTCP training in Pune Municipal Corporation, India, 2010.

Characteristics	Exposed to RNTCPtraining* (n = 136)		Not exposed to RNTCPtraining (n−113)		Total(N = 249)	OddsRatio	(95% CI)
	N	(%)	n	(%)			
**Gender**							
Male	102	(55.1)	83	(44.9)	185	1.08	(0.59–1.99)
Female	34	(53.1)	30	(46.9)	64		
**Medical Qualification**							
MBBS+ & above	29	(39.2)	45	(60.8)	74	2.44	(1.35–4.43)
Non MBBS++	107	(61.1)	68	(38.9)	175		
**Clinical Experience** +++							
≤10 years	51	(54)	44	(46)	95	0.94	(0.55–1.62)
>10 years	85	(55.2)	69	(44.8)	154		
**Relationship with RNTCP** **							
Involved	101	(71.1)	41	(22.9)	142	5.07	(2.84–9.07)
Not involved	35	(32.7)	72	(67.3)	107		
**Clinic hours attending to TB patients** †							
Evening hours	124	(53.4)	108	(46.6)	232	0.42	(0.11–1.47)
No Evening hours	11	(73.3)	4	(26.7)	15		

*Exposure to RNTCP was defined as participating in a RNTCP training module of at least 4 hours at any time from 1999 through 2010.

**Revised National TB Control Programme of India.

+ Allopathy practitioner (Bachelor of Medicine and Bachelor of Surgery).

++ Doctors practicing homeopathy, Ayurveda, Unani.

+++ Number of years in clinical practice after completion of medical training.

† Information not available for two study participants.

Of the 249 participants, 63% (158) indicated that they use microscopy of two sputum specimens for diagnosis of new pulmonary TB. Many providers reported use of additional diagnostic methods for diagnosis of new pulmonary TB as well: 90% (223 out of 249) used chest X-ray, 92% (229/249) used erythrocyte sedimentation rate (ESR), 48% (120/249) used serological tests, and 55% (137/249) used tuberculin skin tests. Odds of reporting investigative methods for diagnosis of pulmonary TB that are in agreement with ISTC guidelines did not differ significantly by exposure to RNTCP training **(** **Table 3** **)**.

**Table 3:** Association between exposure to Public Private Mix (PPM) and self-reported investigation methods used for diagnosis of new pulmonary TB among private providers – Pune Municipal Corporation, India, 2010.

Self-reported choice of investigativeapproach (N = 249)	Exposed to Training*(Exposed, n = 136)	Not exposed to training(Not Exposed, n = 113)	Odds ratio**	(95% CI)
	N (%)	N (%)		
Two Sputum smear microscopy(n = 158)	94 (69%)	64 (57%)	1.71	(0.99–2.98)
Chest X-ray (n = 223)	115 (84%)	108 (96%)	0.25	(0.08–0.74)
Tuberculin Skin test(n = 137)	83 (61%)	54 (48%)	1.7	(1.0–2.9)
Erythrocyte Sedimentation Rate(n = 229)	126 (93%)	103 (91%)	1.2	(0.4–3.3)
Serological tests −IgG/IgM(n = 120)	65 (48%)	55 (49%)	0.9	(0.5–1.6)

*Exposed to RNTCP training- Attended training programme.

**Odds ratio relates to doctors who reported choice of a particular investigation, by history of attendance of CME relative to those who did not attend.

Response rates varied for questions regarding diagnostic and treatment practices, although differences based on RNTCP training did not reach statistical significance based on 95% CI. **(** **Table 4** **)**. PP who had been exposed to RNTCP training tended to adhere to international standards for diagnosis of new pulmonary TB and previously treated pulmonary TB. For new pulmonary TB, 94/136 (69%) of PP who were exposed to RNTCP training adhered to diagnostic standards compared to 64/113 (57%) of providers who were not exposed to RNTCP training (OR 1.7, 95% CI 0.99–2.98). For previously treated pulmonary TB, 99/126 (78%) of PP exposed to RNTCP training adhered to diagnosis standards compared to 69/98 (70%) of providers not exposed to RNTCP training (OR 1.54, 95% CI 0.80–2.96). For diagnosis of extrapulmonary TB, proportions of PP adherent to international standards were similar irrespective of exposure to RNTCP training (Table 3). Associations between RNTCP training and self-report of diagnostic or treatment practices that are in accordance with international standards remained non-significant after stratification by qualification (data not shown).

**Table 4:** Association between exposure of private providers to Public Private Mix (PPM) and self-reported TB clinical treatment and diagnostic practices that are in accordance with international standards-Pune Municipal Corporation, India 2010.

Number of respondents	Exposed to RNTCPtraining*	Responses in agreementwith ISTC**		Odds ratio†	(95% CI)
TB Diagnosis ClinicalScenario		n	%		
Pulmonary TB Case (n = 249)	Exposed (n = 136)	94	69	1.71	(0.99–2.98)
	Not exposed (n = 113)	64	57		
Extra-Pulmonary TB case (n = 247)	Exposed (n = 135)	79	59	0.61	(0.35–1.08)
	Not exposed (n = 112)	78	70		
Previously treated TB case (n = 224)	Exposed (n = 126)	99	79	1.54	(0.80–2.96)
	Not exposed (n = 98)	69	70		
**TB Treatment Clinical** **Scenario**					
New Pulmonary TB Case (n = 249)	Exposed (n = 136)	48	35	1.17	(0.66–2.05)
	Not exposed (n = 113)	36	32		

*Exposed to RNTCP training- Attended training programme.

**International Standards of TB Care.

† Odds ratio relates to doctors who adhered to guidelines, by attendance of training relative to those who did not attend.

For treatment of new cases of pulmonary TB, 48/136 (35%) of PP exposed to RNTCP training adhered to treatment standards compared to 36/113 (32%) of PP without exposure to RNTCP training (OR 1.17, 95% CI 0.66–2.05). Overall, 34% (84/249) of PP reported use of only 4 first line drugs for treatment of new pulmonary TB and 38% (94/249) indicated that in addition to four first line drugs they would use one or more second line anti-TB drugs. Overall, 26% (65/249) of PP reported use of fluoroquinolones for new TB. Use of second line drugs to treat new pulmonary TB was greater for PP who had been exposed to RNTCP training compared to those not exposed, though this association did not reach statistical significance (OR 1.5, 95% CI 0.9–2.7).

## Discussion

In a systematic random sample of private practitioners in an urban district in India, we found serious gaps between self-reported diagnostic and treatment practices and international standards, irrespective of prior RNTCP training. Particularly striking was that only 34% of respondents reported use of the international standard anti-TB treatment regimens. It was also noted that a large proportion (26%) of PP reported addition of one or more second-line anti-TB drugs to the first line regimen. This is concerning because inappropriate use of second-line anti-TB drugs for treatment of drug-sensitive TB may promote the emergence of more complicated forms of drug-resistant TB [9].

After a decade of RNTCP training to promote implementation of ISTC among private providers, just over half of private providers reported exposure to RNTCP training. However, we did find that PP who are involved with RNTCP were more likely to have been exposed to RNTCP training than PP not involved with RNTCP, suggesting that training programmes may be one of the interventions for involving PP in collaboration with the RNTCP.

Though policies and contents of the training changed in context with treatment of smear negative cases, it did not affect the study as management of smear negative TB was not part of the study.

The proportion of private providers who have participated in RNTCP activities was similar to that reported from surveys in Kerala and Delhi, but in these settings 40% and 60% of private providers follow RNTCP or international guidelines, respectively [10]–[11].

In general, we found associations between exposure to RNTCP training and self-reported clinical practices consistent with international standards, though these associations did not meet statistical significance. However, this may be due in part to insufficient sample size and power to detect differences at the levels observed, which differed from our sample size estimates. This finding should be interpreted with some caution; a similar study from another large city in South India, Chennai Corporation, did find a significant improvement in self-reported TB management practices after exposure to Public Private Mix (PPM) activities [12].

In the past, very few private practitioners used sputum microscopy among the initial diagnostic tests for suspected pulmonary TB. [8] We found that 63% of private practitioners report use of sputum microscopy for diagnosis of new pulmonary TB, in accordance with ISTC. Our data on use of smear microscopy are consistent with findings from other recent studies, which report a high proportion of providers using smear microscopy as an initial diagnostic tool [11], [12], [14]. However, a large proportion of private practitioners also report use of other tests which are not recommended by the RNTCP for diagnosis in adults, such as tuberculin skin testing or erythrocyte sedimentation rate (ESR) and serological tests. Dozens of distinct commercial serological tests are marketed in many parts of the world specially in developing countries with weak regulatory systems. [15] Serological diagnostic tests for TB were recently banned in India, which may change this particular practice in the future.

Most studies conducted among PP in India show wide variation in self-reported diagnosis and treatment practices, but these have been limited by a lack of representativeness in the provider sample [12], [13], [15], [16]. This study had several limitations. First, only PP registered with professional organizations were eligible for inclusion, so we were not able to assess the clinical practices of those providers not belonging to professional organizations. Assessment of clinical practices relied on self-report and not all participants provided responses to each clinical scenario, therefore we cannot exclude reporting bias. History of participation in RNTCP was by self-report and we were not able to validate this information. We asked providers about participation in training activities at any time during an eight year period; because of this long recall period we cannot exclude recall bias in reporting of activities. In addition, the duration of time between training of PPs and this study was not taken into account in assessing management practices. Some policies and training content changed over the time period of interest; however the primary RNTCP training module published in 2002 was used throughout the period of interest. We were unable to stratify responses by time since training so could not assess any possible effect of waning knowledge after a prolonged gap since training. Finally, we were not able to measure the extent to which trainings were implemented as intended or to verify their content. What providers reported may not correlate to what they did; substantial differences have been reported between self-reported provider practices and objective practice quality assessments.

Strategies that have been used successfully in India to improve adherence by private practitioners to international standards of care include timely provision of professional support services for retrieval of patients lost to follow up and documentation of treatment services, efficient logistics management, and reinforcement of education in standards of care through one-to-one contact between private practitioners and staff of the national TB control programme [12]. Training and sensitization programmes may need to be tailored to individual providers to address specific gaps in practice and modified to incorporate the choices of private providers.

Despite a decade of effort to train PP, a high proportion of private practitioners in Pune report TB management practices not in accordance with international guidelines. Because private practitioners play a significant role in the diagnosis and treatment of TB, approaches to engage the private sector in India are critical. Training and education should focus on raising the minimum standard of care provided to patients diagnosed and treated in the private sector, and reducing the inappropriate use of second line drugs that could lead to more cases of drug resistant TB.

## Supporting Information

Questionnaire S1 (DOC)
