# Supplementary material for: Tuberculosis Management Practices of Private Practitioners in Pune Municipal Corporation, India
Source: PLoS One. 2014 Jun 4;9(6):e97993. doi: 10.1371/journal.pone.0097993 (PMC4045673; doi:10.1371/journal.pone.0097993)
Supplement: Questionnaire S1 — (DOC) [file pone.0097993.s001.doc]

**Questionnaire S1**

**Section I**

**1. General information-**

A) Practitioners Study Code___________

B) Interviewer Study code: ___________________________

C) Date of interview: ______________________

D) Time of Interview-------------------------

E) Address of PP: ______________________

F) Contact number of PP-------------------------

G) Age: _______________________________

H) Sex: Male/Female

I) Qualifications:

- MBBS / BAMS / BHMS/BUMS/DHMS/DMS/ other __________
- MD( )/ MS (____________)

J) How many years are you in **private practice**? _________________Yrs

**K) Timing** of the clinic -_9 AM to 1 PM

- - - 1. **PM to 5 PM**
      2. **6 PM to 9 PM**
      3. **24 hours**________________________________

L) Are you aware of the Revised National TB Control Program/DOTS Programme (If no move to **Section 2**)- Yes/No

M) If yes how did you come to know? What is source of your information?

a) Programme organized by Government b) Subscription to medical journal

c) Programme organized by Indian Medical association d) Internet

e) Discussions with colleague

N) Are you involved in RNTCP Yes/No (If no move to **3**)

O) If Yes as I) referral doctor II) DOT centre II) DMC III) If Other Specify…..

P) Have you signed MOU-----------------

Q) Have you attended CME on RNTCP - Y/N

R) Have you attended training programme of 4 to 6 hours on RNTCP-Y/N

**Section 2**

3 Kindly Opine on the Case situations mentioned below:

**Case Number -1**

37 years female is complaining of cough since 2 weeks, fever since 7 days and chest pain in Right side of the chest since last 2 days?

1. Do you think investigations are required in this case? Yes / No (If no move to II)

If yes-Please tick the Investigation you advice for patient.

|  | **Please Tick Mark (√)** | **Place of investigation** | **Number if specimen** |
| --- | --- | --- | --- |
| **HB** |  | NA |  |
| **TLC, DLC** |  | NA |  |
| **ESR** |  | NA |  |
| **X-Ray** |  | NA |  |
| **ELISA** |  | NA | NA |
| **Sputum smear examination** |  |  |  |
| **Sputum Culture Examination** |  |  |  |
| **Tuberculin skin test etc.** |  | NA |  |
| **Other –Please specify** |  | | |

1. Do you think treatment is required in this case? –Yes / No

III If No, What is your further advice -___________________________________________

**IV If treatment is required -Please write a prescription** (Drugs, Doses, frequency, duration)

Rx,

**V. If patient shows a report having Sputum Positive** TB what is your Prescription?

| Drugs with doses | Daily or Intermittent | Duration |
| --- | --- | --- |
|  |  |  |

***VI. How would you monitor progress of patient during treatment?***

| **Investigations** | **Please Tick Mark (√) your preference.** |
| --- | --- |
| Clinical examination |  |
| X-ray |  |
| Sputum examinations |  |
| **Other: Please Specify** | |

**Case situation 2**

The above patient completed course of treatment as per your advice above. But after 1 year came with same complaints ie cough for 2 weeks.

I. Do you think **investigations** are required in this case? **Yes / No** (**If no move to II**)

II.If yes- **Please tick the Investigation you advice for *this patient***

|  | **Please Tick Mark (√)** | **Place of investigation** | **Number if specimen** |
| --- | --- | --- | --- |
| **HB** |  |  |  |
| **TLC, DLC** |  |  |  |
| **ESR** |  |  |  |
| **X-Ray** |  |  |  |
| **ELISA** |  |  |  |
| **Sputum smear examination** |  |  |  |
| **Sputum Culture Examination** |  |  |  |
| **Tuberculin skin test etc.** |  |  |  |
| **Other –Please specify** |  | | |

**III. If patient found to be of relapse case of TB what is the Treatment prescribed:**

Rx,

**Case Situation 3**

18 year old male presenting with non-tender swelling on left side of the neck. He also complains of fever and loss of appetite. How would you manage this patient?

**I. Investigations: _________________________________**

***II.. If patient*** is diagnosed as Lymph Node TB how would you treat him?

| **Drugs with doses** | **Daily or Intermittent** | **Duration** |
| --- | --- | --- |
|  |  |  |

***III.*** How do you ensure that patient takes complete treatment?

1. Intensive Counseling and examination during all visit
2. Call him every - 7 days / 15 days /1 month / other please specify frequency ___
3. Keep a record in the clinic/hospital to track his progress If yes How ?
4. Phone call the patient to enquire about his progress
5. Refer the patient to government health centre
6. Other Methods adopted: Please elaborate: _______________________________________________________________________

***IV.* How do you “document/record” details of TB patients on treatment with you?**

__________________________________________________________________________

V.. Who among the following patients are more “at risk” of developing TB?

|  | **Please Tick Mark (√) your preference** |
| --- | --- |
| Malnutrition |  |
| Hypertension |  |
| HIV |  |
| Smokers |  |
| Diabetes Mellitus |  |
| Other | |

**Section 3**

| **Interview of Practitioners involved in RNTCP activities** | **Yes/No** |
| --- | --- |
| Do you have a DOTS **sign board** |  |
| Do you have RNTCP **IEC Material** at present? |  |
| Do you have RNTCP laboratory **referral forms** at present? |  |
| Do you get the **feedback of the patients referred by you** for diagnosis? |  |
| Do you have **sputum containers** at present? |  |
| Do you get **feedback** of the patients referred by you for treatment? |  |
| Did you get any honorarium for provision of DOT? |  |
| **For the practitioners who are not involved /Exposed in RNTCP** |  |
| Are you aware of various RNTCP schemes for involvement in RNTCP? |  |
| Would you like to be a part of RNTCP NGO/ PP schemes? |  |
| If you are not willing may we know the reason? | |
